# A Flying Platform to Investigate Neuronal Correlates of Navigation in the Honey Bee (*Apis mellifera*)

**DOI:** 10.3389/fnbeh.2021.690571

**Published:** 2021-07-20

**Authors:** Benjamin H. Paffhausen, Julian Petrasch, Benjamin Wild, Thierry Meurers, Tobias Schülke, Johannes Polster, Inga Fuchs, Helmut Drexler, Oleksandra Kuriatnyk, Randolf Menzel, Tim Landgraf

**Affiliations:** ^1^Department of Biology, Chemistry and Pharmacy, Institute of Neurobiology, Free University of Berlin, Berlin, Germany; ^2^Dahlem Center for Machine Learning and Robotics, Department of Mathematics and Computer Science, Institute of Computer Science, Free University of Berlin, Berlin, Germany

**Keywords:** honeybee (*Apis mellifera* L.), neuroethology, navigation, mushroom body, naturalistic condition, quad copter, electrophysiology

## Abstract

Navigating animals combine multiple perceptual faculties, learn during exploration, retrieve multi-facetted memory contents, and exhibit goal-directedness as an expression of their current needs and motivations. Navigation in insects has been linked to a variety of underlying strategies such as path integration, view familiarity, visual beaconing, and goal-directed orientation with respect to previously learned ground structures. Most works, however, study navigation either from a field perspective, analyzing purely behavioral observations, or combine computational models with neurophysiological evidence obtained from lab experiments. The honey bee (*Apis mellifera*) has long been a popular model in the search for neural correlates of complex behaviors and exhibits extraordinary navigational capabilities. However, the neural basis for bee navigation has not yet been explored under natural conditions. Here, we propose a novel methodology to record from the brain of a copter-mounted honey bee. This way, the animal experiences natural multimodal sensory inputs in a natural environment that is familiar to her. We have developed a miniaturized electrophysiology recording system which is able to record spikes in the presence of time-varying electric noise from the copter's motors and rotors, and devised an experimental procedure to record from mushroom body extrinsic neurons (MBENs). We analyze the resulting electrophysiological data combined with a reconstruction of the animal's visual perception and find that the neural activity of MBENs is linked to sharp turns, possibly related to the relative motion of visual features. This method is a significant technological step toward recording brain activity of navigating honey bees under natural conditions. By providing all system specifications in an online repository, we hope to close a methodological gap and stimulate further research informing future computational models of insect navigation.

## 1. Introduction

Honey bees are remarkable navigators. Foragers learn to orient in complex environments and perform accurate goal-directed flights in areas several square kilometers in size (Collett, 1996; Menzel and Greggers, 2015). A range of experimental evidence and computational models regarding which strategies bees may employ have been put forward (Srinivasan et al., 1996). Path integration, visual guidance using view memories or structured landmark memories may play a role. However, it is still unknown whether and how those components are combined and at which level of computation they may be available to a navigating bee (Collett, 2019; Webb, 2019). In most animal models, the search for the neural correlates of navigation has made considerable progress through experiments in which the recorded animal was able to move freely in close-to-natural environments (O'Keefe and Nadel, 1979; Bingman and Able, 2002; Hafting et al., 2005; Rubin et al., 2014; Eliav et al., 2021).

In insects, we can identify two main approaches: animals may either move freely in small confined arenas, such that their brain is accessible with wire electrodes or imaging techniques (Jin et al., 2014, 2020; Kim et al., 2017), or they are tethered in virtual reality setups moving stationarily (Harrison et al., 2011; Zwaka et al., 2019). Early evidence showed that bees accept virtual stimuli (Abramson et al., 1996), and virtual reality arenas in which bees can explore artificial environments “afoot” have been established (Schultheiss et al., 2017; Buatois et al., 2018). However, while other insects have been shown to readily fly in virtual environments (Kaushik et al., 2020), so far only one virtual reality arena for bees reported flights just over one minute long (Luu et al., 2011). No neurophysiological data has yet been obtained from bees flying in virtual reality. Recording from neurons using a backpack of miniaturized hardware as proposed in dragonflies (Harrison et al., 2011) is still infeasible due to size and weight constraints in bees. As a result of this technological gap, little is known about the neural correlates of flight navigation in bees.

Substantial previous research in various insect species has identified potential candidate neuropils that may play a role in navigation. Recent work, however, suggests that even minor differences between the connection patterns of different insect species may yield a significantly different functionality of these circuits (Pisokas et al., 2020), underlining the necessity of neurophysiological access to navigating honey bees in flight.

Where should we look for neuronal correlates of navigation? The central complex was found to house neurons essential for sun compass related navigation (Homberg et al., 2011). Body direction cells were found in the cockroach's central complex under conditions that allowed testing of immediate memory effects as they appear under dynamic spatial object-body relations. They thus may play a role in guiding walking trajectories under natural conditions (Varga and Ritzmann, 2016). Ring neurons in the Drosophila central complex were found to code body direction in relation to simulated visual objects (Kim et al., 2017), and these neurons are thought to play a role in the directional component of path integration (Seelig and Jayaraman, 2015). However, the central complex is difficult to access in honey bees. It lies below the mushroom bodies (MBs), another important neuropil that integrates multi-modal sensory input and is involved in memory formation (Menzel, 2014). Particularly in the context of navigation, the MB has been previously hypothesized to store view memories that the navigating insect could match with its current observations (Menzel, 2012; Webb and Wystrach, 2016; Müller et al., 2018; Webb, 2019) and would then continue moving into directions of highest familiarity. Previous work confirmed detrimental effects on higher-order forms of learning (Komischke et al., 2005; Devaud et al., 2007) when interfering with the mushroom body's functioning (Buehlmann et al., 2020; Heinze, 2020; Kamhi et al., 2020). Mushroom body extrinsic neurons (MBENs), neurons at the output of the mushroom body, are likely involved in memory formation and retrieval (Menzel, 2014) and have been successfully recorded in freely walking honey bees (Duer et al., 2015; Paffhausen et al., 2020). Moreover, a subset of MBENs can be targeted precisely under visual control after exposing only a fraction of the brain (Menzel, 2013). This increases the animal's survival rate over extended recording durations, and hence, we here decided to target MBENs. The MBs multimodal and learning-related properties make it a much more suitable target in the context of real-world vs. virtual reality. It seems more likely to trick the central complex with a VR stimulation to process meaningful information related to navigation. The MB, however, has the potential to be more sensitive to the integration of multimodal stimulation. The synchrony, resolution, and comprehensiveness of the real world may be particularly helpful when investigating the involvement of the MB during navigation.

We propose a novel methodology to record neuronal activity from MBENs of honey bees on a quadcopter. The animal can be flown automatically along predefined routes presenting natural stimuli in all sensory modalities. We performed behavioral experiments to verify that bees show flight behavior when tethered on the copter and can integrate visual information perceived on the copter in subsequent episodes of autonomous navigation. Supported by these results, we developed a miniaturized recording system that is capable of amplifying and digitizing neural activity while reducing motor and rotor noise to acceptable levels. In this paper, we specify all system components and show the results of our behavioral experiments. We provide a detailed account of experiments in which we successfully recorded neurophysiological data in flight and present an analysis that confirms that the recorded activity is linked to the sequence of stimuli perceived along the flown routes. This is the first work that proves that this alternative to virtual environments is indeed feasible. By opening all system specifications, code and data, we hope to encourage the community to continue these efforts to identify the neural correlates of navigation in honey bees^1^.

## 2. Methods

### 2.1. Behavioral Experiments

**Figure 1:**
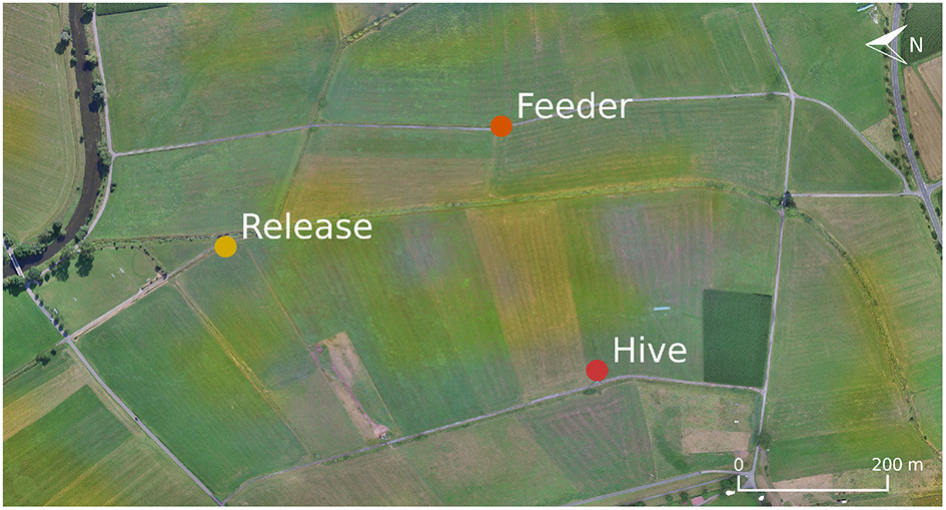
Map of the field site for the behavioral experiments. For the homing experiment, honey bees from the hive (Red) where trained to forage at the indicated feeder location (Orange). Individuals caught at this site were attached to the quadcopter and transported to the release site (Yellow).

Behavioral experiments were conducted in a grassland east of Großseelheim, Germany. A two-frame observation hive was set up at the western border of the field (50° 48' 51.1452" N, 8° 52' 20.9928" E). The field site was rich in visual landmarks, both on the ground (irrigation channels, footpaths, hedges, etc.) and the horizon (see the map in Figure 1 and panoramic images in Supplementary Material).

#### 2.1.1. Do Tethered Bees Show Flight Behavior on a Drone?

**Figure 2:**
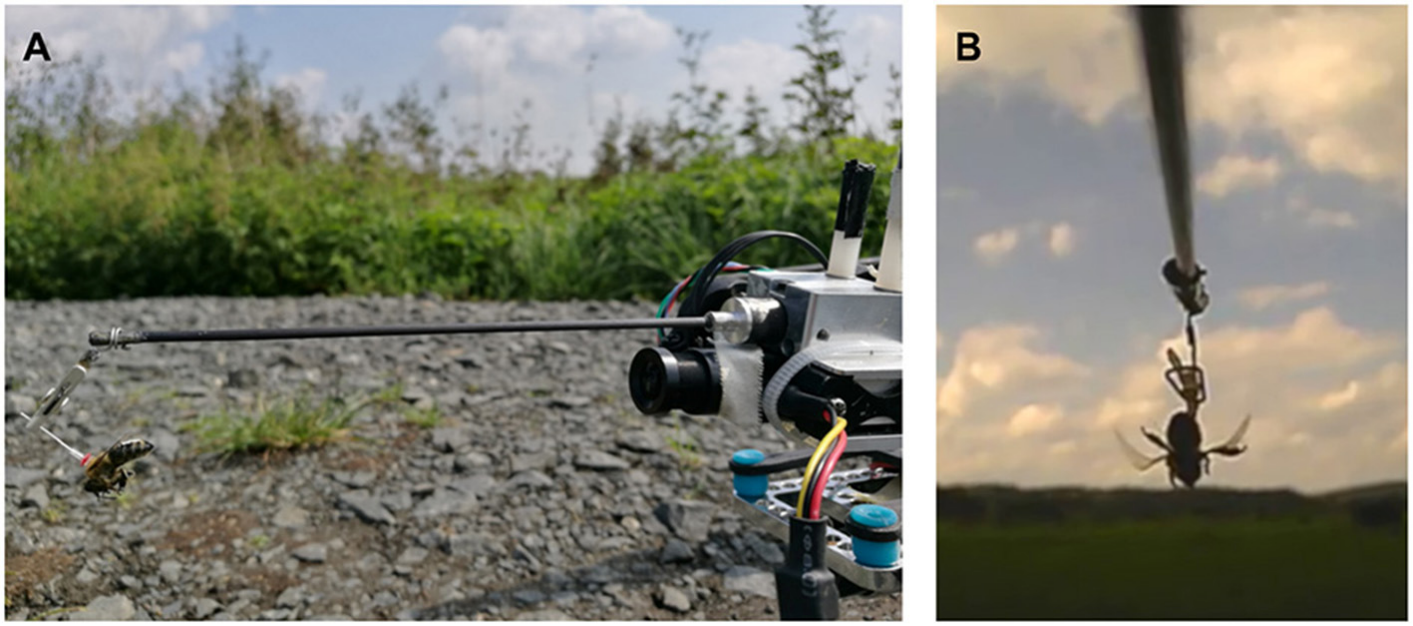
Behavioral field experiments. **(A)** Honey bee attached to a quadcopter via an extension arm. **(B)** Still image of a video recording showing flight behavior defined as continuous wing beating, with raised abdomen and hind legs.

Honey bee (*Apis mellifera*) workers from three groups (hovering—*H*, forward flying—*F*, and control—*C*) were attached to a quadcopter (Matrice 100, DJI, Shenzhen, China) via an extension arm (50 cm in length, see Figure 2). A number tag with a small metal pin was glued to the animal's thorax, and the pin was clipped to the extension arm. The arm positioned the bee such that it had an almost unoccluded view. The copter was placed in the field, and the animal was allowed to grab a light foam ball (~8 mm in diameter) attached to the ground via a string. A camera behind the animal recorded video at 25 Hz to an SD card. At the start of the experiment, the copter lifted off from the ground (groups *H* and *F*), pulling the bee from the foam ball. For the control group *C*, the ball was pulled manually, without any motor activity of the copter. Due to the tarsis reflex, the bees started beating their wings instantaneously. Bees in group *H* were lifted upwards to ~2 m altitude (natural altitude during short foraging trips with a distance of 30 m), with negligible rotatory or horizontal movement. Bees in group *F* were flown forwards, continuously gaining altitude (up to 2 m) and distance to the lift-off point. The copter was controlled manually and brought back after no wing beating was observed anymore or a maximum of one minute of flight time had passed. Flight forward velocity was 10 m/s (natural flight speed observed during radar experiments, Riley et al., 2005). Videos were analyzed after the fact, and the duration of continued wing beating was extracted. Each of the 47 bees was tested with all treatments in randomized order with resting intervals of 1 min.

#### 2.1.2. Homing After Copter Flight

We investigated if bees extract information relevant for homing when being transported on the copter. Bees were trained to a sugar dish 400 m east of their hive (50° 48' 56.25" N, 8° 52' 38.766" E, see Figure 1) and caught after drinking *ad libitum*. A small plastic marker with a metal pin was glued to the number tag they already had affixed to their thorax. The animal was then either clipped to the copter's extension arm (treatment group *T, N* = 54) or put in an opaque box on the top face of the copter (control group *C, N* = 18) such that it could not perceive the flight path visually. The animal was tethered with a small clamp in the box, similar to the mechanism depicted in Figure 2. The procedure took ~1 min. The copter was then started manually, ascending vertically to 15 m altitude, and was then set to reach the target location automatically (400 m north of the feeder location: 50° 49' 6.4632" N, 8° 52' 30.5616" E). Both lift-off and landing procedures were performed manually because automatic lift-off and landing were implemented with a slow rate. Flight velocity was 10 m/s. Upon arrival at the target location, the bee was untethered and released. The time and ID of the bee were noted upon release and arrival at the hive. Some bees landed in the grass shortly after taking off. For these bees, we noted the time they resumed their return flight.

### 2.2. Neuronal Correlates of Navigation

#### 2.2.1. Miniaturized Recording System

**Figure 3:**
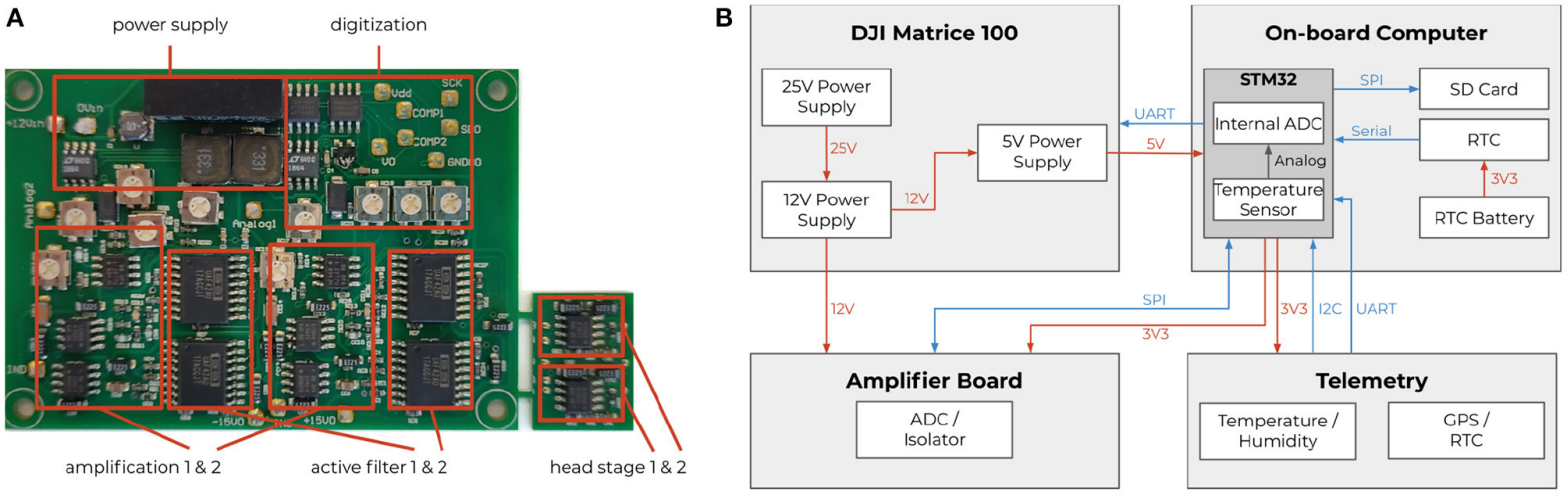
Extracellular amplifier and data flow. **(A)** Photograph of the custom two-channel extracellular amplifier. The design contains two head stages to convert the impedance. They are located close to the bee's head. Each analog signal is then processed separately by amplifiers (amplification factor: 1,000x) and active filters (bandpass: 300 Hz–10 kHz). The resulting analog signals were then digitized by two synchronized delta-sigma analog to digital converters (16 bit, 20 kHz sampling frequency). Two galvanic isolators isolated the digital signals to not pick up any noise from the data storing microcontroller (STM32F4). **(B)** Schematics of the system with a STM32F4 as on-board computer. Diagram of all electrical components of the neurocopter system and the used buses additionally to the components which are parts of the DJI Matrice 100 quadcopter. The blue arrows represent data transfer. The direction of an arrow symbolizes the direction of the information flow. The red arrows show power supplies and their respective voltages. To make the code hardware independent, the STM32 cube hardware abstraction layer was used for hardware access.

To record neural activity from the bee's brain, we developed a lightweight, battery-driven amplifier, and a data acquisition and storage system. The custom solution consisted of a two-channel extracellular amplifier, two analog-digital converters (ADC), and a microcontroller board with an SD card for data storage. The amplifier (see Figure 3) was based on a suitable one-channel amplifier (Budai, 2004). The circuit board (PCB) contained two of those amplifiers, a shared power supply, and two electrically isolated ADCs that were read out simultaneously by a dedicated microcontroller. The head stages were laid out on a separate PCB, located close to the bee. This way, the weak neural signals had to travel only a few centimeters. The electrode bundle (Duer et al., 2015) consisted of two enameled copper wires and a bare silver wire as reference. The reference wire was bent 90° relative to the copper wires, 80 μm above the electrodes' tip, to indicate the desired depth of electrode placement in the brain. The two input channels were measured and amplified in reference to the shared ground electrode. The resulting signals were later subtracted from each other in the digital domain to form a differential pair. The impedance of each electrode was highly dependent on the final recording site, i.e., the surrounding tissue and their electric properties. An offline impedance matching allowed for the most accurate noise cancelation (see section 2.3).

Electric noise reduction was of particular importance due to the disproportionately small voltage and current of the brain signals and the noise emanating from the copter. In-flight, the copter generates strong electric and electromagnetic fields. The plastic rotors generate electric fields by statically charging due to the air friction, and the four motors driving the rotors generate strong electromagnetic fields. Each motor is connected to a motor controller that generates strong switching noises, interferes with the copter's battery voltage, and generates electric field changes. All those influences were considered when the amplifier's power supply was designed and isolators were chosen. The cables transmitting analog signals were particularly susceptible to noise. Copper tape was used to shield all cables from electric field interference. The recorded signals were amplified such that the biological signals were detailed enough for sufficient spike sorting, but the large voltage changes would not saturate the input range of the amplifier (see **Figure 8**). The amplified and filtered (100–20,000 Hz) signals were digitized and read out by a microcontroller board (see Figure 3). This component acquires timestamps from a connected GPS module and stores the data on an SD card. The neural data, therefore, was synchronized to the copter telemetry data.

#### 2.2.2. Quadcopter

**Figure 4:**
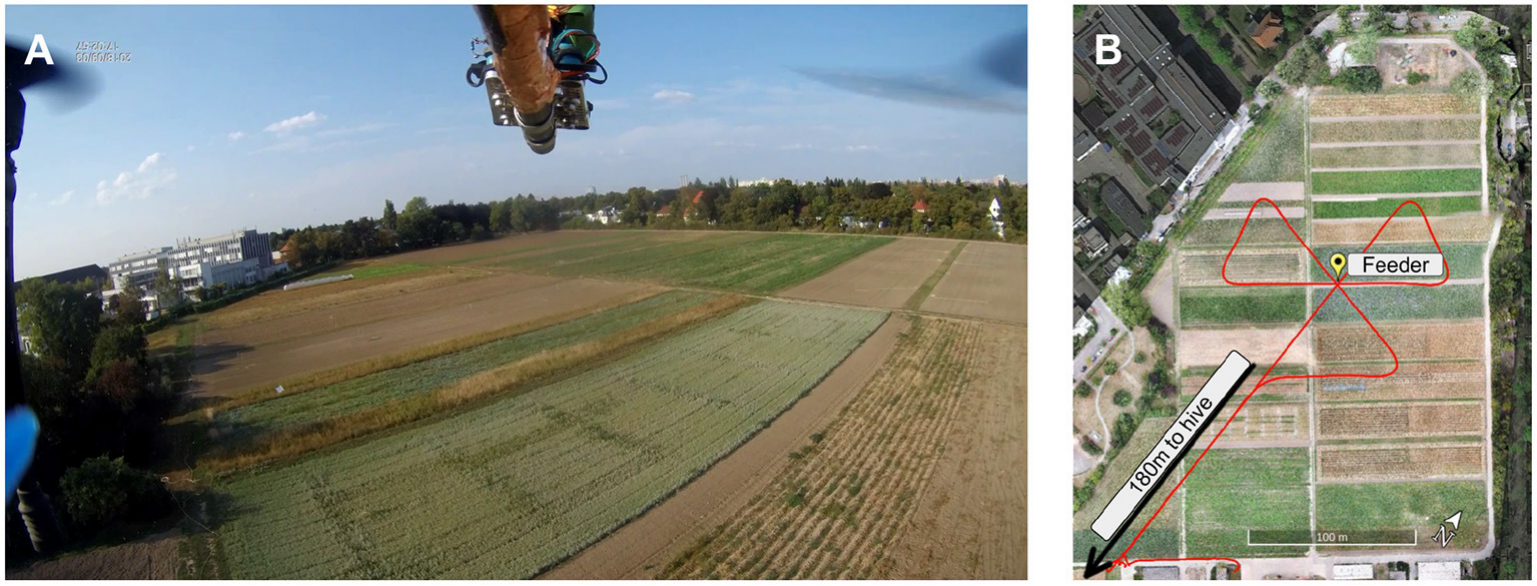
Environment of the navigation experiments. **(A)** View of the onboard video camera. The frame shows the experimental environment while the copter is en route to the feeder (see marked location in **B**). **(B)** Trajectory of the trefoil flight pattern. Flights started at the south-west corner of the field, ~180 m from the hive.

A quadcopter (Matrice 100, DJI, Shenzhen, China) was equipped with the miniaturized recording system and an extension arm to attach the animal and recording equipment. A camera observed the tip of the arm from below and provided a view of the environment (Yi 4k, YI Technologies, Singapore, see Figure 4). The battery case was retracted slightly to balance the weight of the extension arm for best flight stability. A custom metal cage on top of the copter contained the amplifier board. It was shielded with copper tape that was connected to the copter battery's negative terminal. The microcontroller board was located on top of the cage. The extension arm also separated the bee from the motors as far as possible without interfering with the flight properties and the center of mass of the copter. The potential pickup of electromagnetic fields emerging from the motors and propellers is decreased this way. The bee stage was connected to the extension arm by rubber dampeners to reduce vibrations.

#### 2.2.3. Field Site and Photogrammetry

A hive was set up at Free University Berlin (52° 27' 25.3116" N, 13° 17' 45.7584" E), and bees were trained to collect sucrose from a feeder on a field (~50,000 m^2^) at Julius-Kühn-Institute Berlin, Germany (52° 27' 39.7008" N, 13° 17' 48.3288" E). All inflight neurophysiological recordings were conducted at this site.

In the post-experimental data analysis, we studied the link between neural activity and the animal's visual input, reconstructed from the copter's position and a realistic 3-dimensional map of the field site. Prior to the experiments, the field was mapped using photogrammetry from aerial imagery (using a DJI Inspire, Pix4D), resulting in a surface depth map. Due to regulations, we were not allowed to fly over the surrounding areas. We extracted freely available image data (Google Earth) in virtual flyovers for the surrounding field (in total 220 km^2^) and reconstructed the depth map in high-resolution (12 cm/pixel) for a close neighborhood around the field and in low resolution (~4 m/pixel) for a larger surrounding area. The three maps were combined in Blender (Blender Online Community, 2018). This way, the high-res map of the field (resolution: ~13 cm/pixel) provided detailed and up-to-date ground structures, while the two other models provided horizon information.

#### 2.2.4. Experimental Procedure

Honey bees were trained to a feeder on the experiment site, 420 m north of the hive. The feeder (0.5 M sucrose solution) was positioned in the middle of the field (see Figure 4, standing on a bright yellow box (80 cm wide, 35 cm long, and 40 cm high). All bees visiting the site were marked. The marking color changed every day to distinguish how many days a bee was foraging at the site.

**Figure 5:**
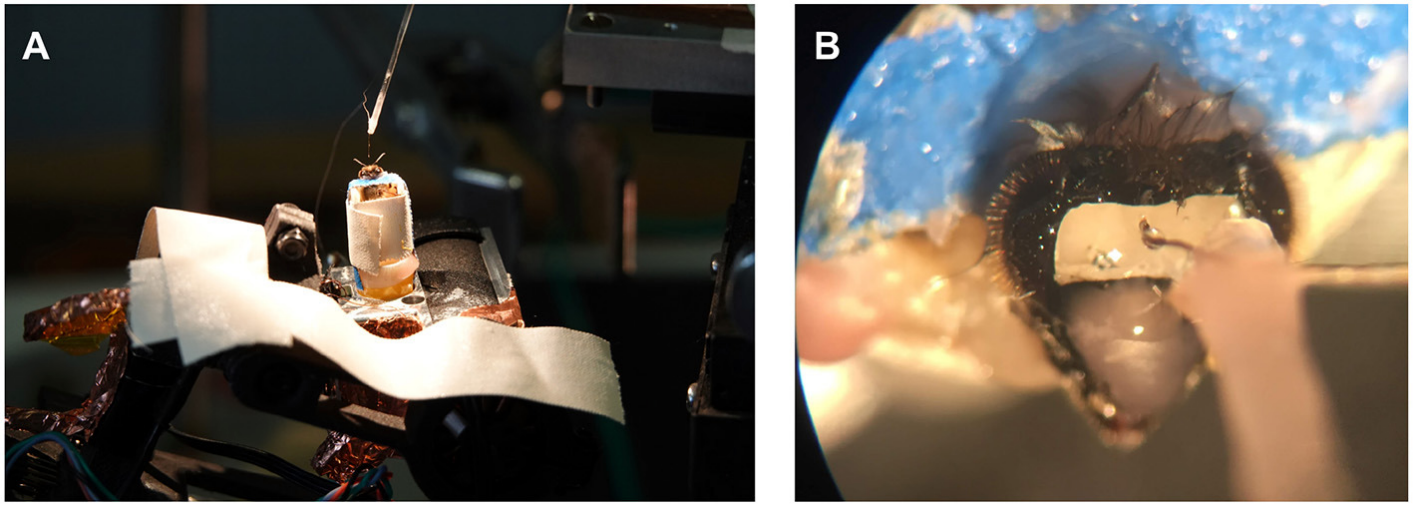
Preparation setup and recording site. **(A)** Preparations for electrophysiological experiments were performed in a controlled environment with the honey bee already attached to the quadcopter. **(B)** A small rectangular incision into the head capsule revealed the bee brain's alpha lobe after the trachea and glands were pushed aside. The electrode bundle visible in the microscope was attached to a micromanipulator with dental wax. Once the electrode bundle was implanted, it was connected to the head stage on the copter via a custom connector jack. Finally, the electrodes and incision were sealed with silicone. Before the animal was moved to the field, the electrode holder was detached to allow for a free anterior view.

In preparation for an experiment, one of the marked honey bees with at least three days of visiting the feeder was caught at the hive. The bee was transferred into a glass vial and anesthetized on ice. The bee, once immobilized, was carefully harnessed in a bee holder with fabric tape and mounted to the recording stage on the copter (see Figure 5). Under stereomicroscopic vision, the head was opened, and the glands and trachea were pushed aside until the alpha lobe was visible (see Figure 5). Two electrodes were implanted into the region of interest. The ground electrode would then rest on the surface of the brain. Once stable neuronal signals were occurring, the electrodes and head aperture were sealed with silicone (Kwik-Sil, WPI, Sarasota, FL, US). The bee and copter were then transported to the field site. A preflight check assured that neural activity was present after light stimulation (acoustic monitoring) and that the recording system was running properly. After the preflight check, the bee was flown automatically on a predefined path resembling a trefoil (see Figure 4). The flight path was chosen such that it includes feeder flyovers from different directions and a flight stretch into the hive's direction. This way, we would be able to explore several hypotheses, e.g., that MBENs respond to familiar views. Bees can see ~300° horizontally on the body plane with low spatial resolution for peripheral ommatidia (Seidl and Kaiser, 1981). The bee holder may thus have blocked a small portion of the posterior view (see Figure 5). Otherwise, the bee had an almost unobstructed view of the environment.

Flights started in the southwest corner of the field, approximately on the line connecting hive and feeder, into the direction of the feeder. The copter lifted off manually to an altitude of 15 m and was then switched to automatic waypoint following. Flight velocity was set to 5.5 m/s. The speed and altitude were chosen because of the legal requirement to maintain line of sight. The copter described a linear path to the feeder and beyond and then executed a right turn ~80 m behind the feeder. The turn reoriented the copter back to the feeder, now facing it from a different bearing. The sequence (feeder overflight and turn) was repeated twice, and the copter then flew back to the start location to repeat this flight pattern until the battery of the copter was too low to continue (3–5 repetitions depending on wind conditions, e.g., head- or tailwinds). The copter was then landed manually at the start site for battery replacement, with consistent rest times between trials. Each time, we downloaded the data to a laptop and then performed the preflight check again. The process was repeated until either the animal died or no spikes could be registered anymore.

### 2.3. Data Analysis

#### 2.3.1. Behavioral Experiments

Statistical hypothesis testing was performed to analyze the behavioral data. For the wing duration experiment (section 2.1.1), a one-sided Mann-Whitney *U*-test was used to test the null hypothesis that there are no significant differences in observed flight behavior duration between the three groups (hovering—*H*, forward flying—*F*, and control—*C*).

For the homing experiment (section 2.1.2), a one-sided Mann-Whitney *U*-test was used to test the null hypothesis that there are no significant differences in the duration of homing flights after the release from the copter between the two groups (treatment group—*T*, control group—*C*).

#### 2.3.2. Neuronal Correlates of Navigation

##### 2.3.2.1. Spike Sorting

The recorded data consisted of two channels of neuronal signals timestamped by GPS signals. The GPS signal was also used to timestamp the telemetry of the copters flight path, which would be used to synchronize the data with sub-millisecond accuracy. The telemetry data was saved with 100 samples per second, including the speed, height, GPS coordinates, acceleration, and orientation of the copter. The data were then merged using the GPS timestamps.

The electrophysiological recordings were analyzed using the Python scientific software stack (Walt et al., 2011; Virtanen et al., 2020). We developed a data processing and spike sorting procedure similar to Quiroga et al. (2004) but adapted to high levels of non-homogeneous noise in the data caused by the motors and rotors of the copter. A robust normalization was applied to both channels separately: *x*_*t*_ = [*x*_*t*_ − median(*X*)]/mad(*X*), where *x*_*t*_ is the amplitude of the signal at time *t* and mad is the median absolute deviation of the signal. The differential of the two recordings was then computed to improve the signal-to-noise ratio in the data. Furthermore, a local robust normalization was applied with a sliding window size of one second to reduce the effect of the time-varying signal-to-noise ratio caused by the quadcopter's motors and rotors on the quality of the extracted signal.

Spikes were then extracted using thresholding. A robust estimate of the standard deviation was calculated as *n* = median(*X*)·*k* (*k* = 1.4826). The threshold for spike detection was set to *Thr* = 4·*n* (Quiroga et al., 2004). Spike positions were then extracted using local minima detection on the thresholded data.

For each detected spike, a window around the peak of the signal of length 1.44 ms was extracted for spike sorting. Haar wavelet coefficients were calculated using PyWavelets (Lee et al., 2019). The dimensionality of these features was reduced using the PCA implementation of scikit-learn such that each remaining feature explains at least one percent of the variance of the wavelet coefficients (Pedregosa et al., 2011). Anomaly detection was performed using the Local Outlier Factor (Breunig et al., 2000) on the PCA features, and detected outliers were not used in further analyses. Spikes were then clustered using the HDBSCAN algorithm (McInnes et al., 2017) on the PCA features using a minimum cluster size of 100.

To increase the method's sensitivity in periods of high noise (e.g., during acceleration of the quadcopter), for each detected neuron, the median spike shape was determined, and the sliding Pearson correlation of this shape with the normalized input signal was computed. The spike detection steps were then repeated on the correlation coefficient, i.e., a threshold was computed, and local minima beyond this threshold were detected. This pattern matching spike detection increased the number of detected non-outlier spikes from 13,861 to 17,106 in a recording of ~14 min.

Spike trains were binned in intervals of 100 ms, and spike rates were calculated as the sum of detected peaks during each interval. For the visualization of the rates in **Figure 8**, a rolling mean with a window size of 3 s was used to smoothen the trajectory.

##### 2.3.2.2. Autocorrelation of Spike Rates

The trefoil path was repeated multiple times per flight, and it seems possible that the neuronal signals reflect these repetitions, irrespective of which sensory properties the units we record from encode. To verify that hypothesis, we calculated the Pearson correlation coefficient of the spike rate time series that corresponds to a single repetition of the trefoil trajectory in a sliding window over the whole flight's recording. If the bee's brain signals reflect the repetitive flight patterns, we expect to see peaks denoting the beginning of every trefoil pattern.

##### 2.3.2.3. Realistic Model of the Honey Bee Compound Eye

**Figure 6:**
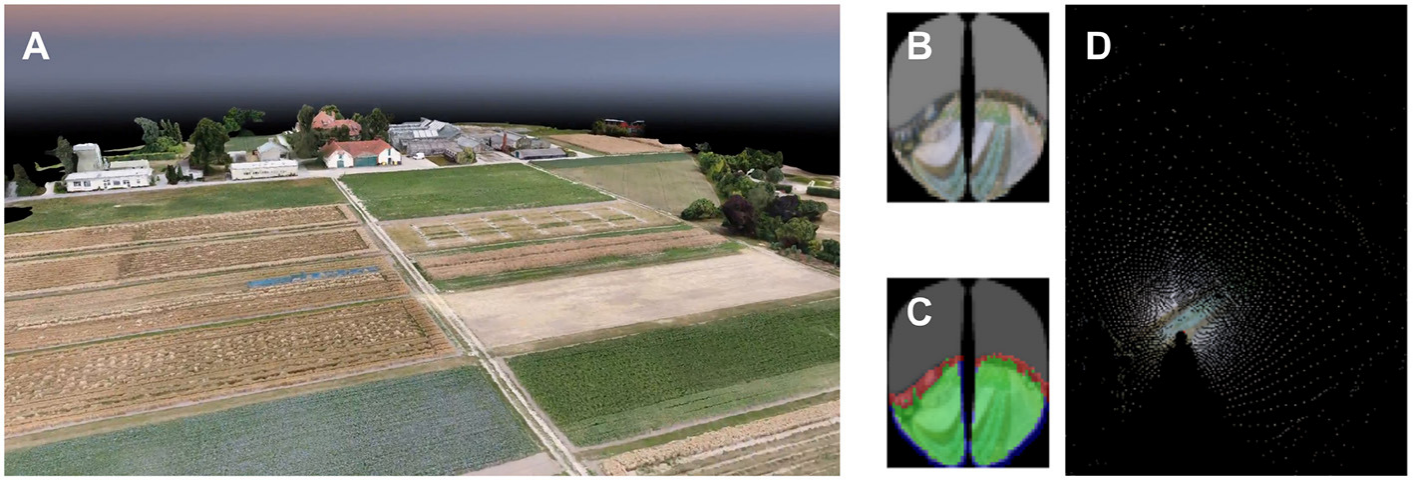
Mapping of a honey bee's vision at one position. **(A)** 3D rendering from the photogrammetric model of the experimental landscape. **(B)** The reconstructed honey bee's perception of the field. To draw this perspective onto a map, the location of each pixel in the photogrammetric model is calculated. **(C)** Only pixels that show a part of the field are used for mapping, which are marked as green in image Red pixels show parts of the environment outside the field. They are not used for mapping as well as blue pixels which lead to artifacts after mapping. **(D)** Ray casting is used to model the locations in the field perceived by the individual and placed on a map of the field. Each mapped pixel is assigned the same color as in the bee image.

In the data analysis, the copter telemetry data (GPS and compass readings) were used to reconstruct the flight path in the 3-D map of the environment. We previously published a software package to reconstruct bees' visual perception (Polster et al., 2019). These bee views mimicked the field of view of the compound eye and the distribution and sampling properties of individual ommatidia. For each 3D position and orientation in the virtual environment, the software casts rays for individual ommatidia and provides a sample of the texture color at the intersection with the 3D model (see Figure 6). To explore whether specific ground or horizon structures may have given rise to repeatable spike activity, we used the software to project spike rates back to the virtual surface.

## 3. Results

### 3.1. Forward Motion Induces Tethered Flight

**Figure 7:**
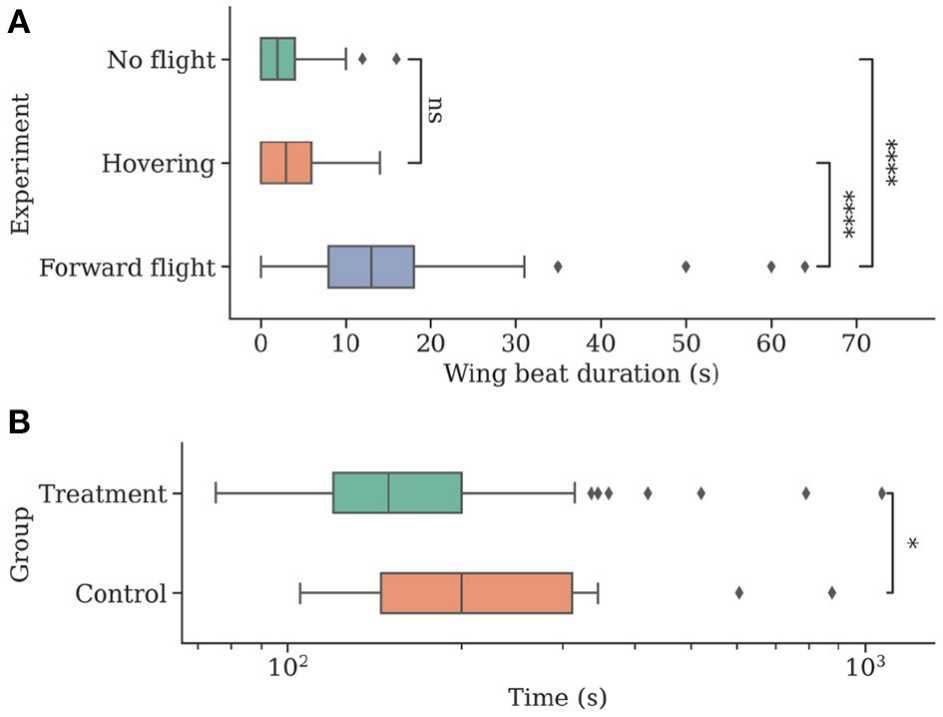
Individuals show more natural flight behavior while being attached to a flying drone and can navigate faster when they can perceive their environment while being transported to a new location. **(A)** The total time of wing beating on the ground, in stationary flight, and during forward movement of the drone. Wing beat behavior occurs for longer durations during a forward movement of the drone (*N* = 47). **(B)** Individuals were caught at a feeding site and replaced to a new location using the drone before being released (*N* = 54). Bees in the control group (*N* = 18) were contained in an opaque container and could not visually perceive their environment during flight. Return times to the hive were measured, and control bees were found to take significantly longer to return.

The forward flight group *F* showed significantly longer wing beating compared to both control and hover groups (median [min, max]; group *F*: 13 s [0 s, 64 s]; *F* vs. group *C*: 2 s [0 s, 16 s], U = 279.5, *P* < 0.001; *F* vs. group *H*: 3 s [0 s, 14 s]; U = 468, *P* < 0.001). Groups *C* and *H* did not differ significantly (U = 2329, *P* = 0.089). See Figure 7 for boxplots of the data.

### 3.2. Copter Transfer Allows Faster Homing

Bees in the treatment group *T* returned home after a significantly shorter amount of time (median [min, max]; group *T*: 149.5 s [75 s, 1,070 s]; *T* vs. group *C*: 200 s [105 s, 875 s]; U = 326, *P* = 0.019). See Figure 7 for boxplots of the data.

### 3.3. Recording Neural Activity Is Feasible on a Flying Copter

Before using the new recording system on the copter, we tested its functionality with artificial signals and signals from a honey bee brain under laboratory conditions. Activity from the same source was recorded with both the copter's amplifier system and a commercial system (amplifier: EXT, npi, Tamm, Germany; digitization: 1401micro, Cambridge Electronics Design, Cambridge, UK). We found no significant differences in the data obtained by these two systems when comparing spikes from bee brains as well as sweeping through frequencies generated artificially.

**Figure 8:**
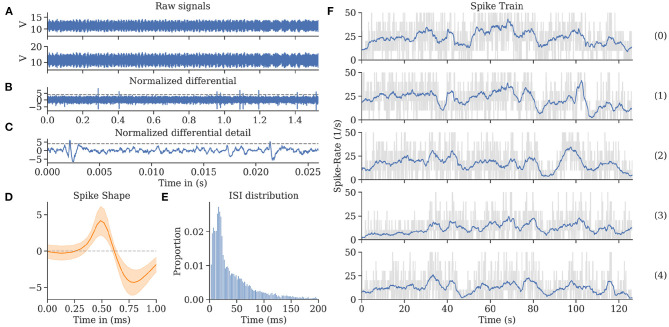
Spike train evaluations for bee A. **(A)** The neuronal activity recorded during a flight from two channels exhibited high noise levels, most of which was eliminated by subtracting the two channels in the digital domain. **(B)** The differential raw trace shows spikes above residual noise. **(C)** Recording from **(B)** zoomed in on two individual spikes (exceeding the horizontal threshold potential). The recording was spike sorted to extract single-unit activity. **(D)** Spike shape template of units shown in **(C)**. **(E)** Inter-spike interval (ISI) distribution showing a single mode at 21 ms and very few instances below 5 ms. **(F)** Spike rate over time per flight (see Figure 4). Each graph represents a repetition of a continuous flight path from start to finish for the same trefoil trajectory. The repetitions share similar features synchronized to the time (and therefore place) of the flown path.

In-flight, we successfully recorded uninterrupted single-unit activity from MBENs for multiple repetitions of the flight trajectory. The electrodes picked up significant amounts of EM noise produced by motor controllers, motors, and propellers. We observed that the noise levels differed between channels, probably due to differences in impedance. However, the differential recording allowed removing much of it when carefully adjusting the respective digital gain factor for one of the channels. For each experiment, the factor was set manually after the fact. Once this tuning was complete, spike shapes emerged. The amplitude of the monopolar input channels was around 100 times larger than the resulting spikes from the differentiated channel. These recordings were then sorted. We calculated interspike intervals and confirmed that the refractory period of 4 ms was rarely undercut, indicating the successful sorting of single spike sources. For more details, see Figure 8.

### 3.4. Neuronal Activity During Flight Is Repeatable

Experiments with neuronal recordings on the copter took place during the fall of 2018 and summer of 2019. Starting with around 200 animals, we successfully implanted electrodes into ~50 animals, all producing neural activity upon light stimulation. However, only 10 animals continued to provide spike data after transport to the field, and only three of these recordings passed the post-experiment quality control check. Half of the recordings showed too much noise over the entire flight list with doubtful spike sorting results, and two recordings were excluded due to variable signal-to-noise ratios throughout the experiment.

**Figure 9:**
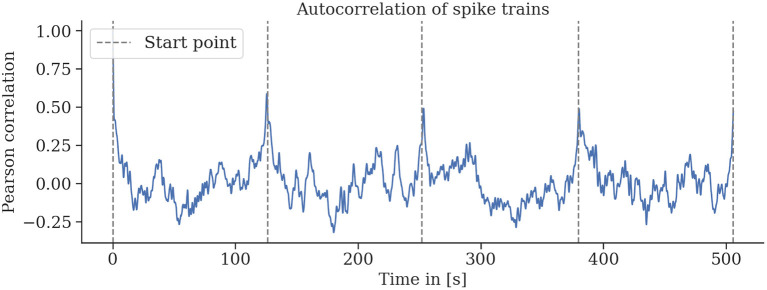
Spike rates are strongly autocorrelated for multiple repetitions of the same flight trajectory. Sliding-window autocorrelations were computed for all rounds of six (a–f) flights. For all flights, particularly for flights a, b, and d, strong correlations of spike activity were observed for several rounds of the same flight trajectory. Gaps between rounds and the starting and landing periods were removed, and the sliding Pearson correlations were computed. Gray lines indicate the start of a round.

The neural recordings are consistent for repeated environmental stimuli. We find strong autocorrelation of the spike rates for single trefoil flight patterns in individual bees (see Figure 9). This indicates a relationship between the phase of the trajectory and the spike rate.

**Figure 10:**
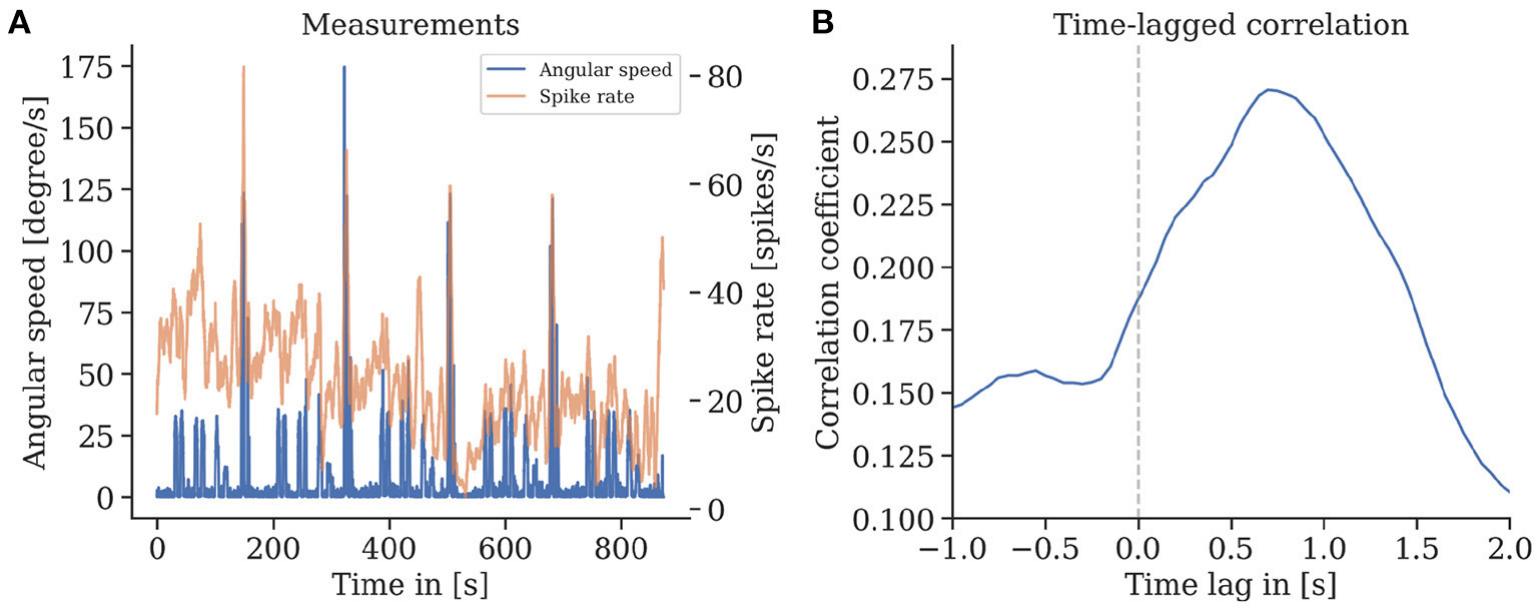
**(A)** Spike rate of mushroom body output neurons of an individual bee and angular velocity during flight. **(B)** A strong time-lagged correlation between spike rate and rotational speed (yaw) was found with a lag of 0.7 s (Pearson's r 0.27, *p* ≪ 0.001).

**Figure 11:**
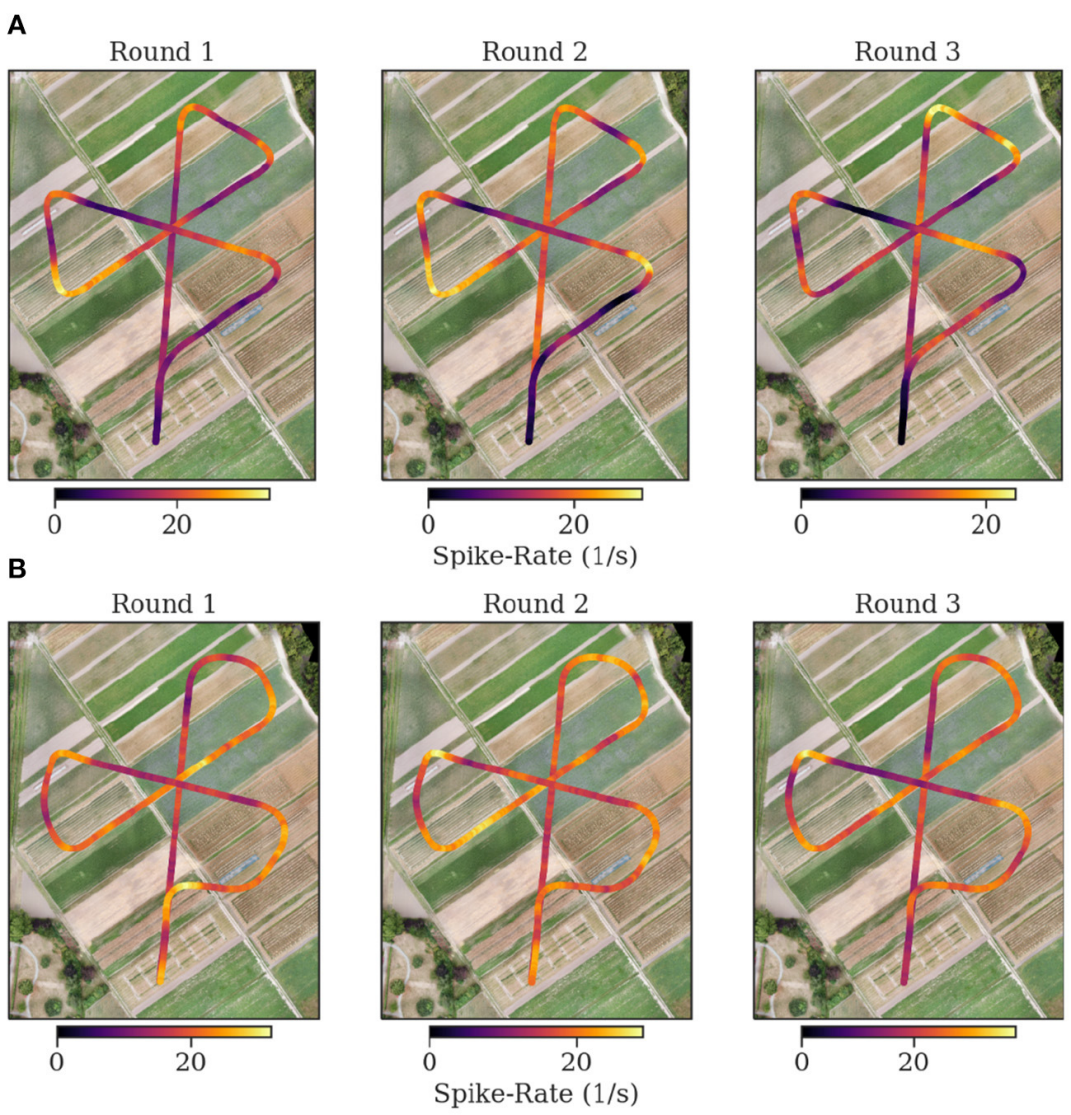
Flight trajectories and spike rate. Each plot shows the spike rate as false color on top of the coordinates of the flown trajectory. The plots are in consecutive order for the first three flights of each bee [**(A)** Bee A, **(B)** Bee B]. The following flights are depicted in the supplementary. At the center of the flower formation, the trained feeder is located. The spike rate in Bee B is more heterogeneous and higher at the corners. The flown corners in the experiments of bee B are sharper than for bee A.

We found that episodes with high spike rates coincide with turning maneuvers (see Figure 10), though high spike rates do not exclusively appear in turns, and some turns do not show higher spike activity. These findings are consistent for repetitions of the same trajectory in one animal but also between individuals. We found a strong correlation of the spike rates with the copter's turning velocity at a latency of 0.7 s. In some recordings, straight flight paths showed spike rate variations as well, yet we did not find any explanation for this behavior (see Figure 11).

**Figure 12:**
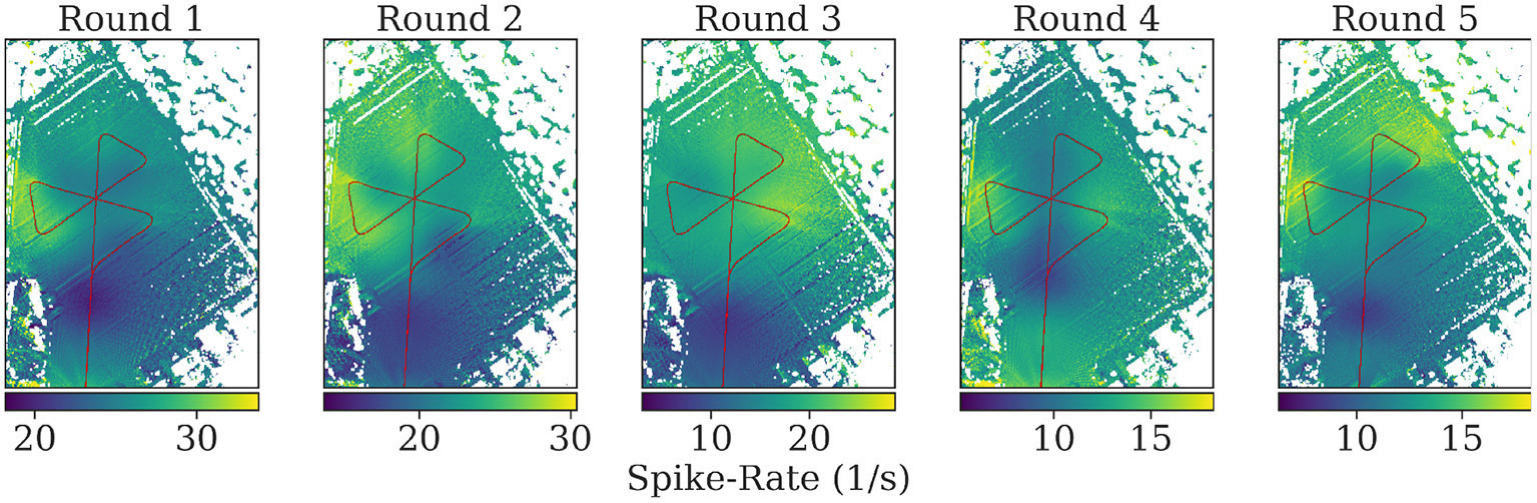
Mapping of the spike rates on the field of view of the individuals during flights. At each position, all pixels in the field of view of the bee on the map are assigned the corresponding spike rate value at that time. The mean is calculated of pixels with multiple assigned spike rate values during mapping. Turns were excluded to highlight spike activity during parts of the rounds without high angular velocity. See the Supplementary Material for the mapped spikes rates including turns.

Visual inspection of the spike rates revealed no apparent correlation to the bee's spatial relation toward the feeder or the hive. We used a model of the bee compound eye to map the spike rate back to the map of the area for each position along the flight path (see Figure 12). While blobs of activity are visible on the resulting maps, they are likely due to single bursts and not due to distributed activity summing up over repeated overflights.

The mapping of spike rate activity to the field of view of the individuals in general revealed regions associated with high spike rates that were varying over multiple repetitions of the same trajectory, even within one individual. Interestingly, even when excluding the turns in this mapping, the regions near the turning maneuvers tended to show the strongest activity (see Figure 11). We found no clear evidence of consistent associations of spike rate with specific landmarks in the data analyzed here.

## 4. Discussion

We propose a novel methodology in the search for the neural correlates of navigation in bees. In contrast to reproducing realistic conditions in virtual environments, we propose moving the lab to the field. While this approach comes with its own challenges, we show that recording neural activity from MBENs in honey bees is feasible on a quadcopter in flight.

We miniaturized the recording hardware, and substantially reduced motor noise picked up by the electrodes with various strategies, from grounding and shielding to differential recordings and respective hardware design decisions.

Those bees that survived the implant and were transported to the field survived multiple repetitions of the flight trajectory. The bees that entered analysis showed no baseline shifts indicating electrode movement nor subsequent loss of units. The electrophysiological data are of high quality, and the spikes and their properties are close to those recorded under lab conditions (Zwaka et al., 2019).

While continuous wing-beating behavior was observed for only a fraction of natural navigational flight durations, forward flight induces longer wing beating compared to the control groups. It is possible that tethered bees, even those that do not fly, perceive their environment as indicated by their significantly shorter homing flights as compared to compromised vision.

Data from three successful neurophysiological experiments may not be conclusive evidence that bees fully retrieve their navigational experience when tethered on a copter, the reproducible neural activities during their trefoil paths, however, suggest that MBENs encode visual features possibly related to the environment. The most prominent correlation we found confirms earlier findings of body turning encoded in MBENs of the cockroach (Mizunami et al., 1998). A similar relationship was found during flight turns in cockroaches (Guo and Ritzmann, 2013). The spike rate correlation could also be related to non-visual stimuli like antennal deflection or changes in inertia.

Before continuing these recording experiments, a few additional key challenges have to be overcome. We need to increase the success rate (currently only 5%) and survival time of the animal. The success rate under laboratory conditions varies between 30% in bees mounted to a tube (Filla and Menzel, 2015) and 10% in bees moving stationary in a virtual environment (Zwaka et al., 2019). The recording electrodes require improvement, particularly the ground electrode that appears to transfer too much mechanical stress onto the brain. One failure case may be attributed to the silicon we used to seal the head access window. In some animals, the signal quality decreased over the time the silicon was set to dry, and we suspect the electrode position to have changed due to shrinkage of the silicone seal. A strong seal that limits electrode movement also throughout the copter flights appears as a crucial element in increasing recording quality.

To reduce post-experiment rejection rates (so far at 70%), we need to further improve the signal-to-noise ratio. On the one hand, this could be accomplished with better shielding and adaptive impedance matching for the channel subtraction. On the other, the assessment procedure that determined whether spike magnitudes are sufficient would benefit from a realistic simulation of anticipated copter noise in the lab.

The main problem for interpreting the results is that spike rates can only be meaningfully compared within the same animal due to potential differences in the neuronal connectivity and electrode implant locations between individuals. Unfortunately, only a finite amount of data can be recorded from one individual, making the interpretation of the results difficult. It may be possible to assess the exact recording location via imaging techniques. To improve the repeatability of the implant and reduce variability in the resulting signals, the electrode production, insertion, and sealing process could be automated in future studies.

An important question to be addressed more accurately in future work will be to relate the localization of the recording electrodes to subsets of MBEN or brain structures such as the central complex. Extracellular recording techniques come with the unavoidable limitation of spatial location. Therefore, the preparatory steps during the selection of the recorded neurons become extremely important. Technical improvements that allow extending the recording time substantially will help characterize the selected neurons physiologically by probing batteries of more complex stimulus conditions before the preparation is fixed to the copter. So far, we selected for stable responses to simple movement stimuli before the bee was fixed to the copter. Thus, it is not surprising that the MBENs analyzed here correlate with turning motions.

Our data analysis includes the reproduction of the bee's visual perception using a three-dimensional map created before the experiments. In contrast to recording synchronized video approximating the field of view of honey bees directly on the copter, our approach drastically reduces the amount of data recorded in each experimental run. However, our model of the honey bee vision using a photogrammetric model of the environment can not simulate the dynamic nature of vegetation, celestial cues, and weather conditions.

Bees have shown flight behavior in a virtual reality setup (Luu et al., 2011), on average even longer than on our copter, despite lacking realism and completeness of the stimulation. While the experimental protocols are not comparable between this and our study, a question still remains for both the drone and the VR approach in general: do bees require a closed feedback channel, i.e., some control over their sensory input, for prolonged flight? Closed-loop bee flight in virtual arenas has not been accomplished yet, possibly due to a lack of realistic multimodal stimulation. Still, while the drone approach offers exactly that, it comes with the challenge of sensing the bee's desired change in body pose under much more noisy conditions—likely a challenge the lab approach may overcome more readily. Why then continue developing the copter system? A likely use-case in the future may be the verification of specific results concerning navigation and neuronal correlates that emerged from VR setups or other lab based experimentations. Functionally relevant claims from such experiments could be put to the test by our method. A verification of results from VR experiments should be valid even with low numbers of bees if the results are consistent. On the other hand, open-loop VR experiments can now investigate whether similar repeatable neuronal activity as shown here can be found in virtual trefoil flights of harnessed bees as well. We will gladly share all relevant data for this comparison (3D map, flight paths and neural recordings).

Our system complements the toolkit for studying the neural correlates of natural navigation in bees. While future developments of lab-based setups may need to focus on a realistic, multimodal reproduction of the environment, drone-based setups are confronted with more complex control tasks. Since, to our knowledge, there is not yet a virtual reality system capable of recording brain activity in flying bees, our system can serve as an alternative starting point. To encourage the continuation of this effort, we are sharing this proof of concept, as presented here, in its entirety with the community.

## Data Availability Statement

The datasets and codes presented in this study can be found in this online repository: https://github.com/BioroboticsLab/neuronal_correlates_honeybee_navigation.

## Author Contributions

BP, RM, and TL conceived of the idea. BP and JPe developed and tested the miniaturized recording system. JPe and TL conceived hardware additions to the copter. BP, JPe, and TM conducted the wing beat duration experiment. TL, BW, and RM conducted the homing experiment. JPe, BP, OK, HD, TL, and IF conducted the neurophysiological experiments. JPe and TM created the drone maps. BW, TS, JPe, BP, and JPo analyzed the data. BP, BW, TL, and RM wrote the manuscript. All authors contributed to the article and approved the submitted version.

## Conflict of Interest

The authors declare that the research was conducted in the absence of any commercial or financial relationships that could be construed as a potential conflict of interest.

## Footnotes

^1^Code and schematics: www.github.com/BioroboticsLab/neuronal_correlates_honeybee_navigation.

## References

[B1] Abramson C. I. Buckbee D. A. Edwards S. Bowe K. (1996). A demonstration of virtual reality in free-flying honeybees: *Apis mellifera*. Physiol. Behav. 59, 39–43. 10.1016/0031-9384(95)02023-3 8848488

[B2] Bingman V. P. Able K. P. (2002). Maps in birds: Representational mechanisms and neural bases. Curr. Opin. Neurobiol. 12, 745–750. 10.1016/S0959-4388(02)00375-6 12490268

[B3] Blender Online Community (2018). Blender- a 3d Modelling and Rendering Package. Blender Online Community.

[B4] Breunig M. M. Kriegel H.-P. Ng R. T. Sander J. (2000). LOF: identifying density-based local outliers. ACM SIGMOD Rec. 29, 93–104. 10.1145/335191.335388

[B5] Buatois A. Flumian C. Schultheiss P. Avarguès-Weber A. Giurfa M. (2018). Transfer of visual learning between a virtual and a real environment in honey bees: the role of active vision. Front. Behav. Neurosci. 12:139. 10.3389/fnbeh.2018.00139 30057530 PMC6053632

[B6] Budai D. (2004). Ultralow-noise headstage and main amplifiers for extracellular spike recording. Acta Biol. Szeged. 48, 13–17.

[B7] Buehlmann C. Wozniak B. Goulard R. Webb B. Graham P. Niven J. E. (2020). Mushroom bodies are required for learned visual navigation, but not for innate visual behavior, in ants. Curr. Biol. 30, 3438–3443.e2. 10.1016/j.cub.2020.07.013 32707069

[B8] Collett T. S. (1996). Insect navigation en route to the goal: multiple strategies for the use of landmarks. J. Exp. Biol. 199, 227–235. 10.1242/jeb.199.1.227 9317693

[B9] Collett T. S. (2019). Path integration: how details of the honeybee waggle dance and the foraging strategies of desert ants might help in understanding its mechanisms. J. Exp. Biol. 222. 10.1242/jeb.205187 31152122

[B10] Devaud J.-M. Blunk A. Podufall J. Giurfa M. Grünewald B. (2007). Using local anaesthetics to block neuronal activity and map specific learning tasks to the mushroom bodies of an insect brain. Eur. J. Neurosci. 26, 3193–3206. 10.1111/j.1460-9568.2007.05904.x 18028113

[B11] Duer A. Paffhausen B. H. Menzel R. (2015). High order neural correlates of social behavior in the honeybee brain. J. Neurosci. Methods 254, 1–9. 10.1016/j.jneumeth.2015.07.004 26192327

[B12] Eliav T. Maimon S. R. Aljadeff J. Tsodyks M. Ginosar G. Las L.. (2021). Multiscale representation of very large environments in the hippocampus of flying bats. Science 372:6545. 10.1126/science.abg4020 34045327

[B13] Filla I. Menzel R. (2015). Mushroom body extrinsic neurons in the honeybee (*Apis mellifera*) brain integrate context and cue values upon attentional stimulus selection. J. Neurophysiol. 114, 2005–2014. 10.1152/jn.00776.2014 26224779 PMC4579295

[B14] Guo P. Ritzmann R. E. (2013). Neural activity in the central complex of the cockroach brain is linked to turning behaviors. J. Exp. Biol. 216, 992–1002. 10.1242/jeb.080473 23197098

[B15] Hafting T. Fyhn M. Molden S. Moser M.-B. Moser E. I. (2005). Microstructure of a spatial map in the entorhinal cortex. Nature 436, 801–806. 10.1038/nature03721 15965463

[B16] Harrison R. R. Fotowat H. Chan R. Kier R. J. Olberg R. Leonardo A.. (2011). Wireless neural/EMG telemetry systems for small freely moving animals. IEEE Trans. Biomed. Circ. Syst. 5, 103–111. 10.1109/TBCAS.2011.2131140 23851198

[B17] Heinze S. (2020). Visual navigation: ants lose track without mushroom bodies. Curr. Biol. 30, R984–R986. 10.1016/j.cub.2020.07.038 32898495

[B18] Homberg U. Heinze S. Pfeiffer K. Kinoshita M. el Jundi B. (2011). Central neural coding of sky polarization in insects. Philos. Trans. R. Soc. B Biol. Sci. 366, 680–687. 10.1098/rstb.2010.0199 21282171 PMC3049008

[B19] Jin N. Landgraf T. Klein S. Menzel R. (2014). Walking bumblebees memorize panorama and local cues in a laboratory test of navigation. Anim. Behav. 97, 13–23. 10.1016/j.anbehav.2014.08.013

[B20] Jin N. Paffhausen B. H. Duer A. Menzel R. (2020). Mushroom body extrinsic neurons in walking bumblebees correlate with behavioral states but not with spatial parameters during exploratory behavior. Front. Behav. Neurosci. 14:590999. 10.3389/fnbeh.2020.590999 33192371 PMC7606933

[B21] Kamhi J. F. Barron A. B. Narendra A. (2020). Vertical lobes of the mushroom bodies are essential for view-based navigation in Australian myrmecia ants. Curr. Biol. 30, 3432–3437.e3. 10.1016/j.cub.2020.06.030 32707060

[B22] Kaushik P. K. Renz M. Olsson S. B. (2020). Characterizing long-range search behavior in Diptera using complex 3D virtual environments. Proc. Natl. Acad. Sci. U.S.A. 117, 12201–12207. 10.1073/pnas.1912124117 32424090 PMC7275712

[B23] Kim S. S. Rouault H. Druckmann S. Jayaraman V. (2017). Ring attractor dynamics in the Drosophila central brain. Science 356, 849–853. 10.1126/science.aal4835 28473639

[B24] Komischke B. Sandoz J.-C. Malun D. Giurfa M. (2005). Partial unilateral lesions of the mushroom bodies affect olfactory learning in honeybees *Apis mellifera* L. Eur. J. Neurosci. 21, 477–485. 10.1111/j.1460-9568.2005.03879.x 15673446

[B25] Lee G. R. Gommers R. Waselewski F. Wohlfahrt K. O'Leary A. (2019). PyWavelets: A Python package for wavelet analysis. J. Open Source Softw. 4:1237. 10.21105/joss.01237

[B26] Luu T. Cheung A. Ball D. Srinivasan M. V. (2011). Honeybee flight: a novel “streamlining” response. J. Exp. Biol. 214, 2215–2225. 10.1242/jeb.050310 21653815

[B27] McInnes L. Healy J. Astels S. (2017). hdbscan: Hierarchical density based clustering. J. Open Source Softw. 2:205. 10.21105/joss.00205

[B28] Menzel R. (2012). The honeybee as a model for understanding the basis of cognition. Nat. Rev. Neurosci. 13, 758–768. 10.1038/nrn3357 23080415

[B29] Menzel R. (2013). In search of the engram in the honeybee brain, in Handbook of Behavioral Neuroscience, Vol. 22, eds Menzel R. Benjamin P. R. (Berlin: Elsevier), 397–415. 10.1016/B978-0-12-415823-8.00029-0

[B30] Menzel R. (2014). The insect mushroom body, an experience-dependent recoding device. J. Physiol. 108, 84–95. 10.1016/j.jphysparis.2014.07.004 25092259

[B31] Menzel R. Greggers U. (2015). The memory structure of navigation in honeybees. J. Compar. Physiol. A 201, 547–561. 10.1007/s00359-015-0987-6 25707351

[B32] Mizunami M. Weibrecht J. M. Strausfeld N. J. (1998). Mushroom bodies of the cockroach: their participation in place memory. J. Compar. Neurol. 402, 520–537. 10.1002/(SICI)1096-9861(19981228)402:4<520::AID-CNE6>3.0.CO;2-K 9862324

[B33] Müller J. Nawrot M. Menzel R. Landgraf T. (2018). A neural network model for familiarity and context learning during honeybee foraging flights. Biol. Cybern. 112, 113–126. 10.1007/s00422-017-0732-z 28917001

[B34] O'Keefe J. Nadel L. (1979). The hippocampus as a cognitive map. Behav. Brain Sci. 2, 487–533. 10.1017/S0140525X00064256

[B35] Paffhausen B. H. Fuchs I. Duer A. Hillmer I. Dimitriou I. M. Menzel R. (2020). Neural correlates of social behavior in mushroom body extrinsic neurons of the honeybee *Apis mellifera*. Front. Behav. Neurosci. 14:62. 10.3389/fnbeh.2020.00062 32372927 PMC7186758

[B36] Pedregosa F. Varoquaux G. Gramfort A. Michel V. Thirion B. Grisel O.. (2011). Scikit-learn: machine learning in Python. J. Mach. Learn. Res. 12, 2825–2830.

[B37] Pisokas I. Heinze S. Webb B. (2020). The head direction circuit of two insect species. eLife 9:e53985. 10.7554/eLife.53985 32628112 PMC7419142

[B38] Polster J. Petrasch J. Menzel R. Landgraf T. (2019). Reconstructing the visual perception of honey bees in complex 3-D worlds. arXiv:1811.07560 [q-bio] [Preprint]. arXiv: 1811.07560.

[B39] Quiroga R. Q. Nadasdy Z. Ben-Shaul Y. (2004). Unsupervised spike detection and sorting with wavelets and superparamagnetic clustering. Neural Comput. 16, 1661–1687. 10.1162/089976604774201631 15228749

[B40] Riley J. R. Greggers U. Smith A. D. Reynolds D. R. Menzel R. (2005). The flight paths of honeybees recruited by the waggle dance. Nature 435, 205–207. 10.1038/nature03526 15889092

[B41] Rubin A. Yartsev M. M. Ulanovsky N. (2014). Encoding of head direction by hippocampal place cells in bats. J. Neurosci. 34, 1067–1080. 10.1523/JNEUROSCI.5393-12.2014 24431464 PMC6608343

[B42] Schultheiss P. Buatois A. Avarguès-Weber A. Giurfa M. (2017). Using virtual reality to study visual performances of honeybees. Curr. Opin. Insect Sci. 24, 43–50. 10.1016/j.cois.2017.08.003 29208222

[B43] Seelig J. D. Jayaraman V. (2015). Neural dynamics for landmark orientation and angular path integration. Nature 521, 186–191. 10.1038/nature14446 25971509 PMC4704792

[B44] Seidl R. Kaiser W. (1981). Visual field size, binocular domain and the ommatidial array of the compound eyes in worker honey bees. J. Compar. Physiol. 143, 17–26. 10.1007/BF00606065

[B45] Srinivasan n. Zhang n. Lehrer n. Collett n. (1996). Honeybee navigation en route to the goal: visual flight control and odometry. J. Exp. Biol. 199(Pt 1), 237–244. 10.1242/jeb.199.1.237 9317712

[B46] Varga A. Ritzmann R. (2016). Cellular basis of head direction and contextual cues in the insect brain. Curr. Biol. 26, 1816–1828. 10.1016/j.cub.2016.05.037 27397888

[B47] Virtanen P. Gommers R. Oliphant T. E. Haberland M. Reddy T. Cournapeau D.. (2020). SciPy 1.0: fundamental algorithms for scientific computing in Python. Nat. Methods 17, 261–272. 10.1038/s41592-019-0686-2 32015543 PMC7056644

[B48] Walt S. v. d. Colbert S. C. Varoquaux G. (2011). The NumPy array: a structure for efficient numerical computation. Comput. Sci. Eng. 13, 22–30. 10.1109/MCSE.2011.37

[B49] Webb B. (2019). The internal maps of insects. J. Exp. Biol. 222(Pt Suppl. 1):jeb.188094. 10.1242/jeb.188094 30728234

[B50] Webb B. Wystrach A. (2016). Neural mechanisms of insect navigation. Curr. Opin. Insect Sci. 15, 27–39. 10.1016/j.cois.2016.02.011 27436729

[B51] Zwaka H. Bartels R. Lehfeldt S. Jusyte M. Hantke S. Menzel S.. (2019). Learning and its neural correlates in a virtual environment for honeybees. Front. Behav. Neurosci. 12:279. 10.3389/fnbeh.2018.00279 30740045 PMC6355692

