# Supplementary material for: A Flying Platform to Investigate Neuronal Correlates of Navigation in the Honey Bee (Apis mellifera)
Source: Front Behav Neurosci. 2021 Jul 20;15:690571. doi: 10.3389/fnbeh.2021.690571 (PMC8329708; doi:10.3389/fnbeh.2021.690571)
Supplement: Supplementary file 1 [file Data_Sheet_1.PDF]

Supplementary Material

1 SUPPLEMENTARY TABLES AND FIGURES

1.1 Figures

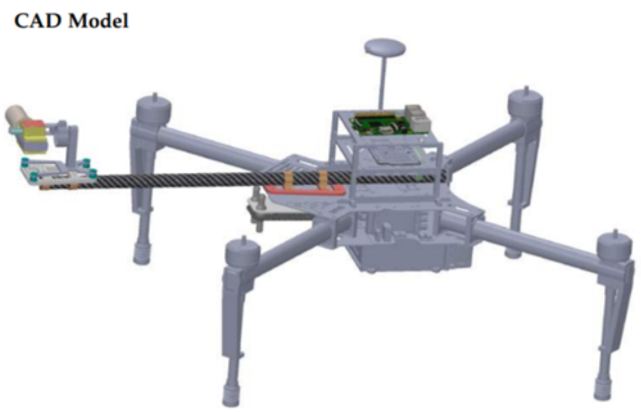

Figure S1. CAD model of the quadcopter with miniaturized electrophysiological recording system.

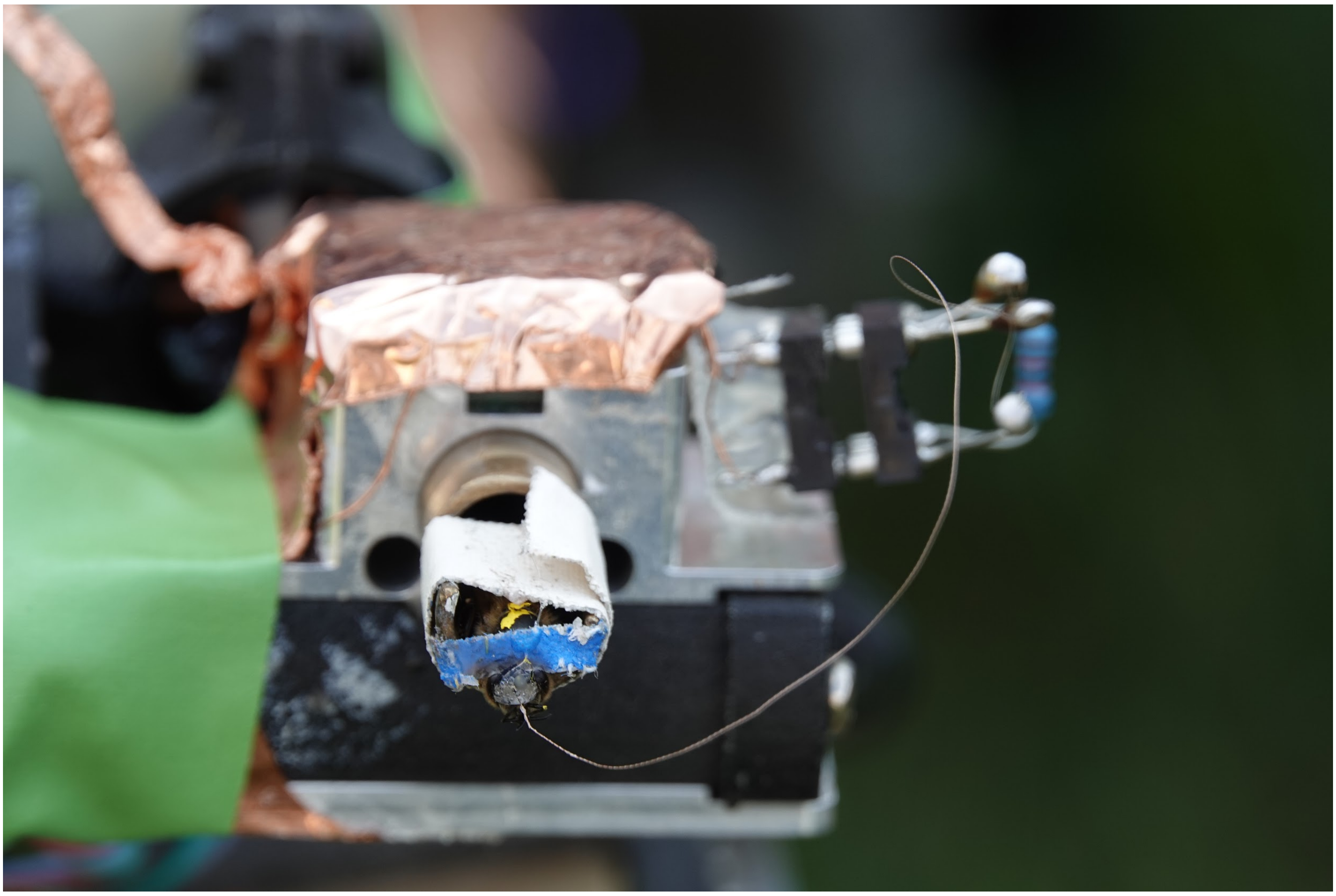

Figure S2. Honey bee mounted on miniaturized recording system prior to experiment.

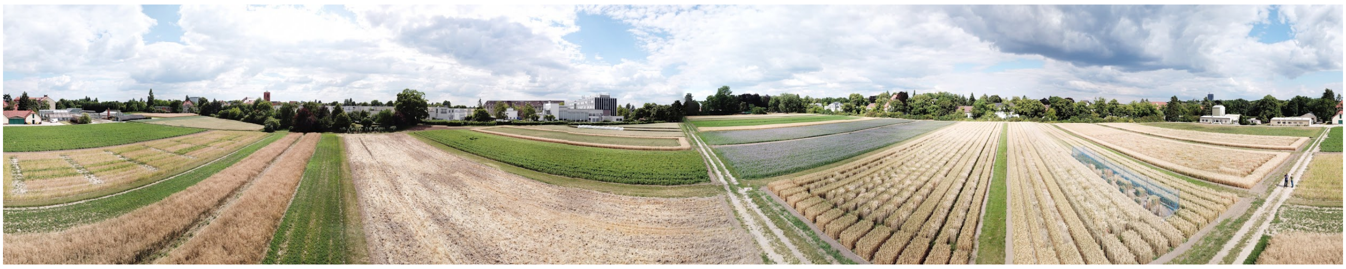

Figure S3. Panoramic view of the navigation experimental field site.

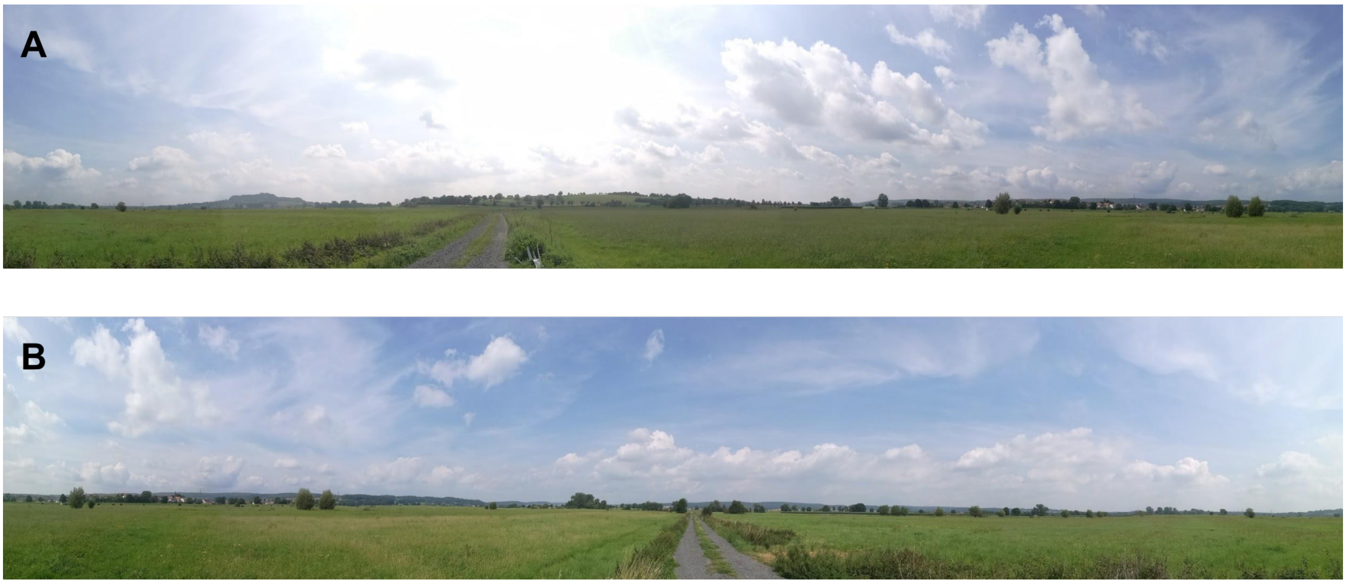

Figure S4. Panoramic view of the behavior experimental field site.

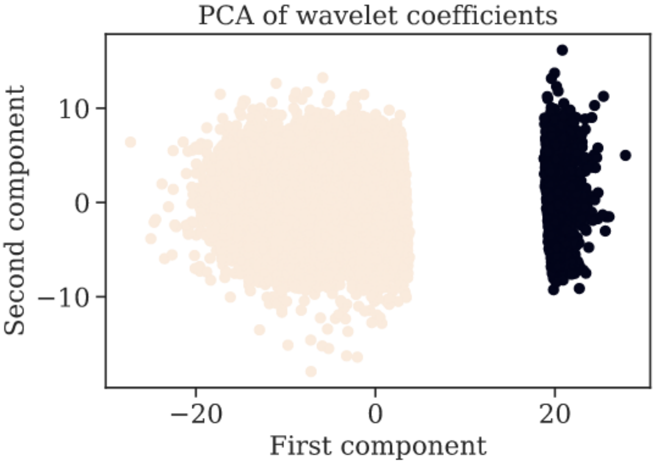

Figure S5. PCA of wavelet coefficients for spike sorting of recordings from bee A.

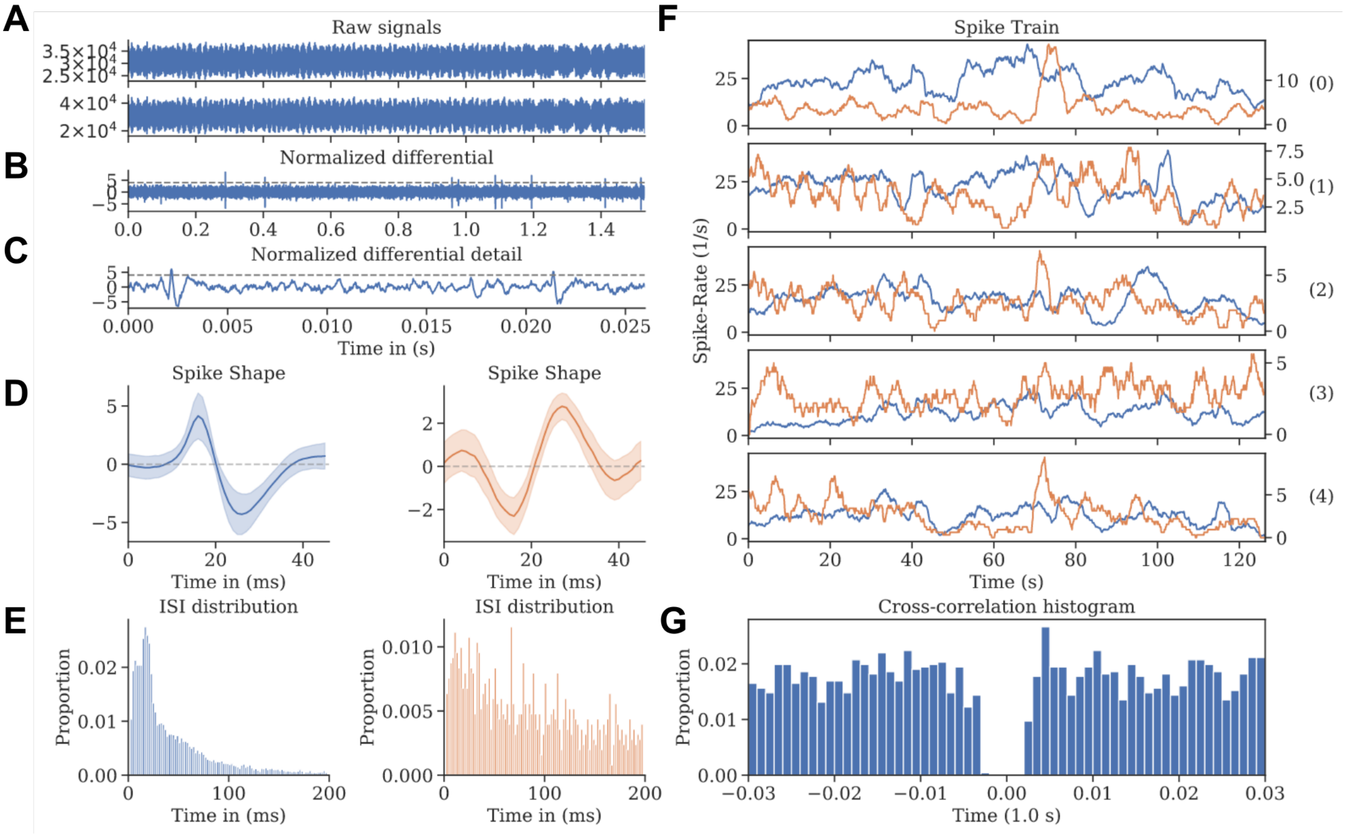

Figure S6. Spike sorting and spike rates for Bee A. The recording was spike sorted to extract the neuronal single units. **D** Spike shape template of **C**. **E** Inter spike interval analysis shows peak at 21 ms and very little ISIs below 5 ms pointing to a single unit. **F** Spike trains over time per flight round. **G** Cross correlation histogram of spikes from the two units shown in **C**.

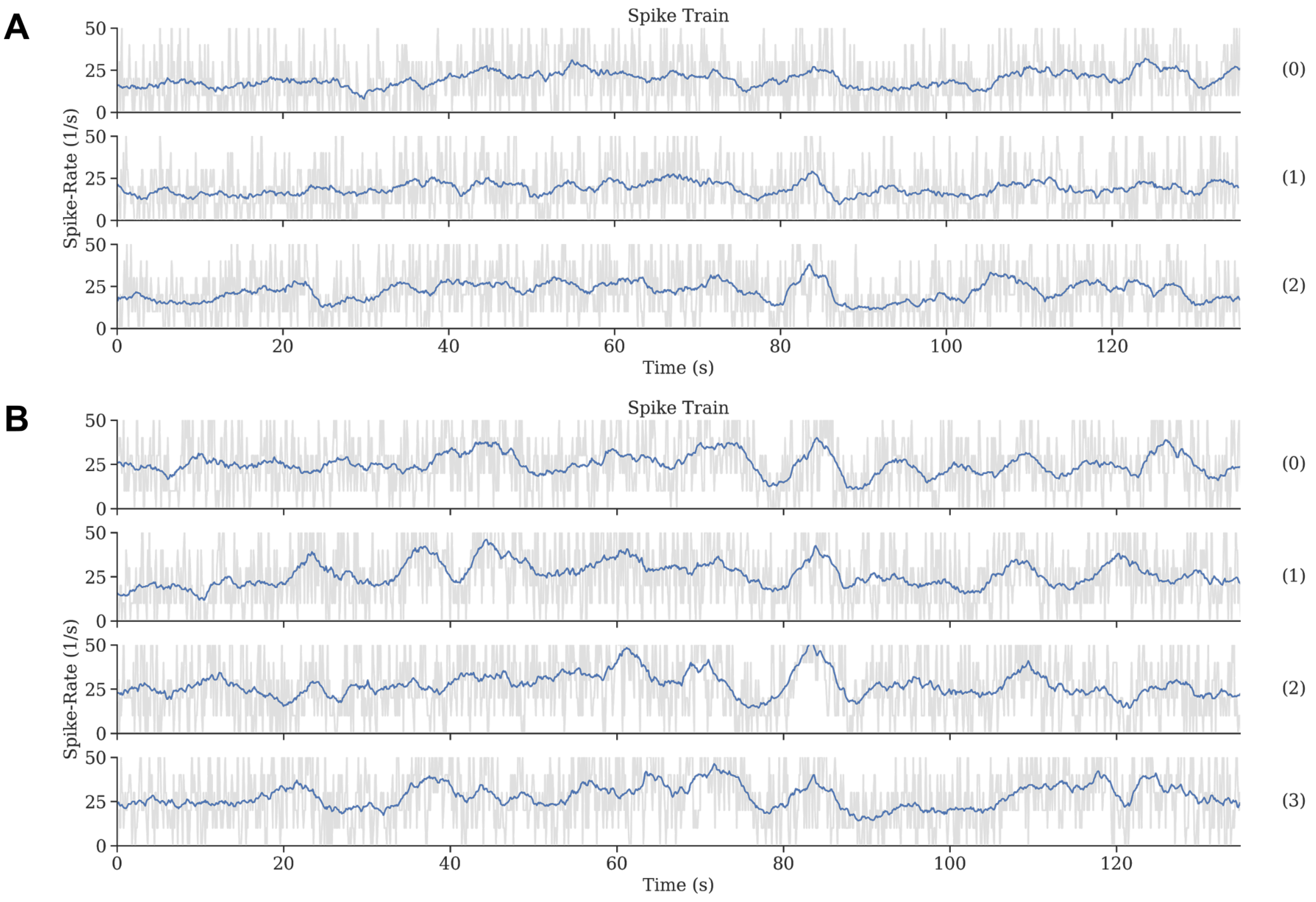

Figure S7. Spike rates from two batteries with several rounds of the repeated trajectory for bee B.

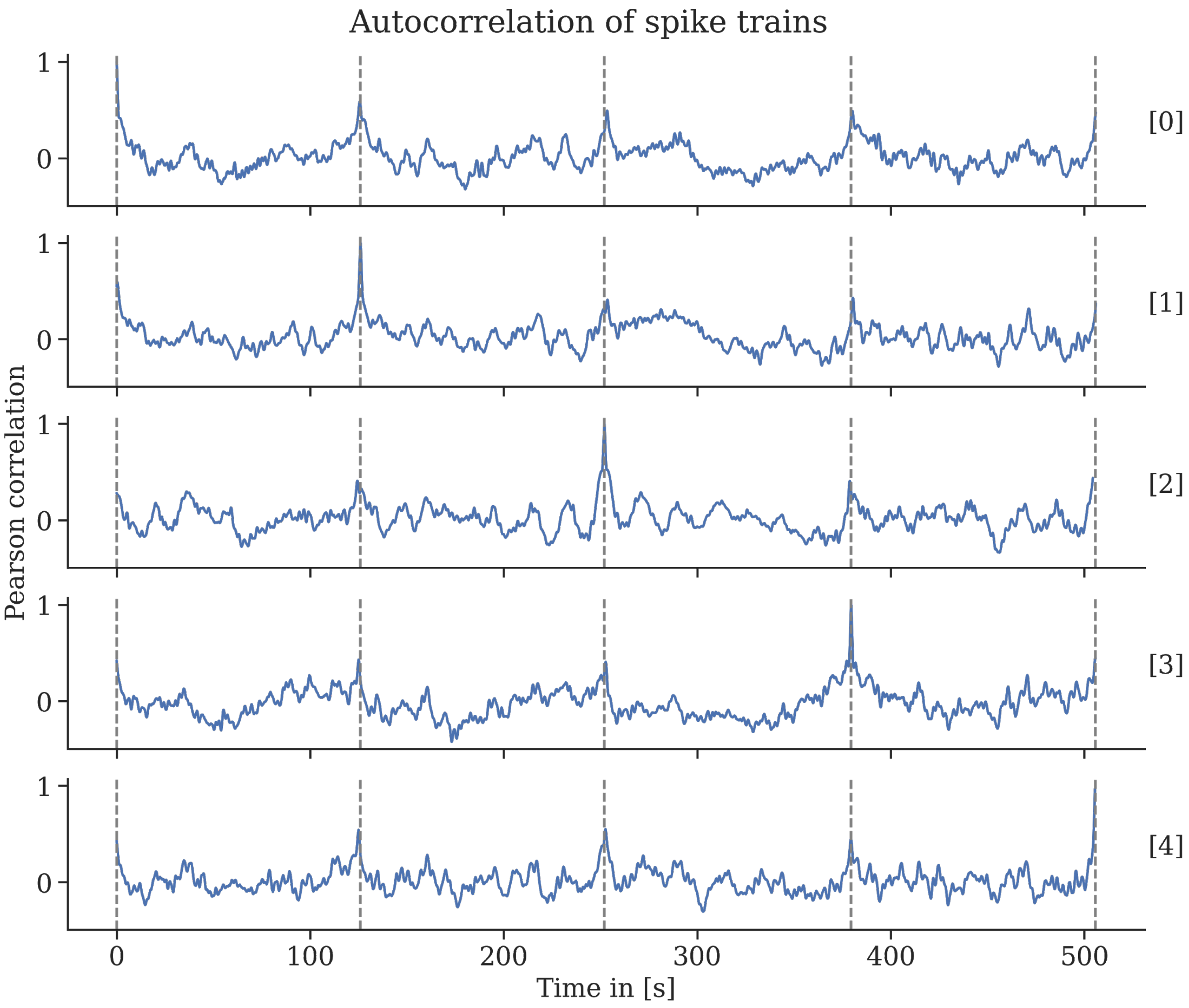

Figure S8. Sliding window autocorrelations for all combinations of repetitions of the flight trajectory for bee A. In each row, one repetition of the flight trajectory is correlated with all other repetitions.

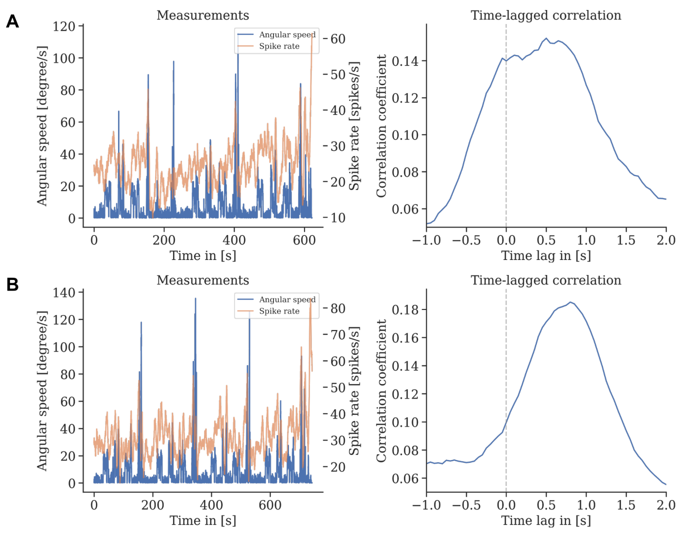

Figure S9. Time-lagged correlation of spike rate and angular speed for bee B. Data from two flights each with several repetitions of the same trajectory. The battery of the copter was exchanged between the flights.

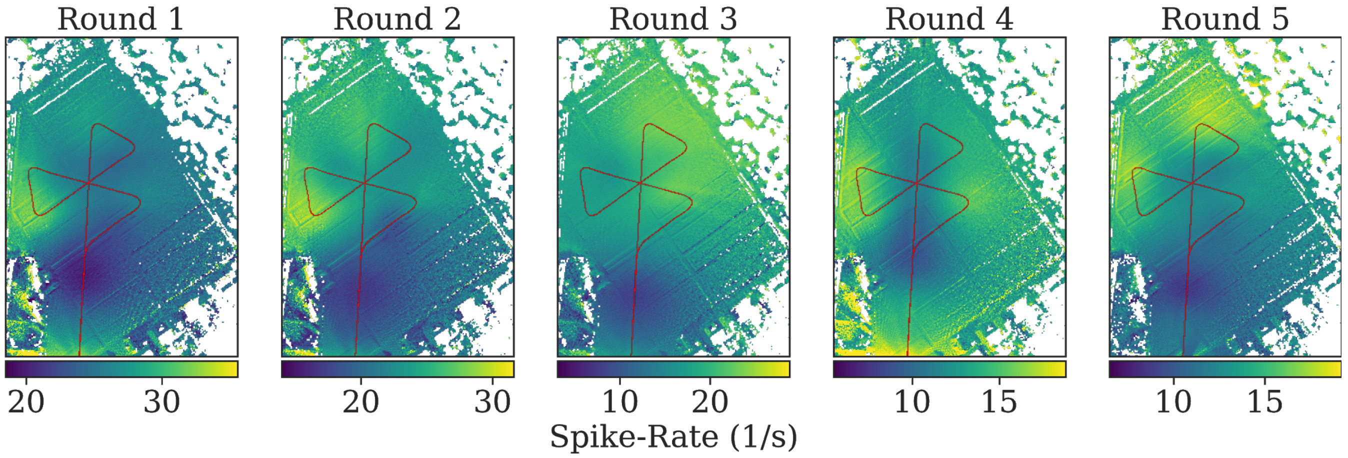

Figure S10. Mapped spike rates during repetitions of the flight trajectory of bee A, including turns with high angular velocity.

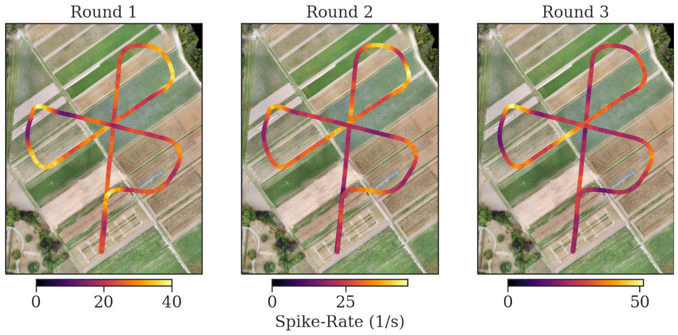

Figure S11. Spike rates projected on map for the first three repetitions from bee B (second battery).
